# Supplementary figures and images for: Ndc1 drives nuclear pore complex assembly independent of membrane biogenesis to promote nuclear formation and growth
Source: eLife. 2022 Jul 19;11:e75513. doi: 10.7554/eLife.75513 (PMC9296133; doi:10.7554/eLife.75513)

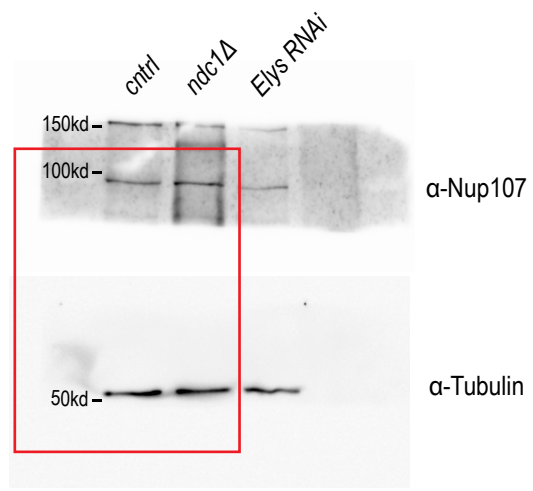

Supplement: Figure 3—figure supplement 1—source data 2. [file elife-75513-fig3-figsupp1-data2.pdf]

**A**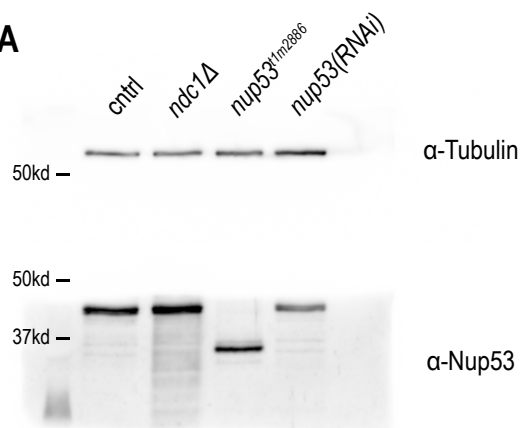**B**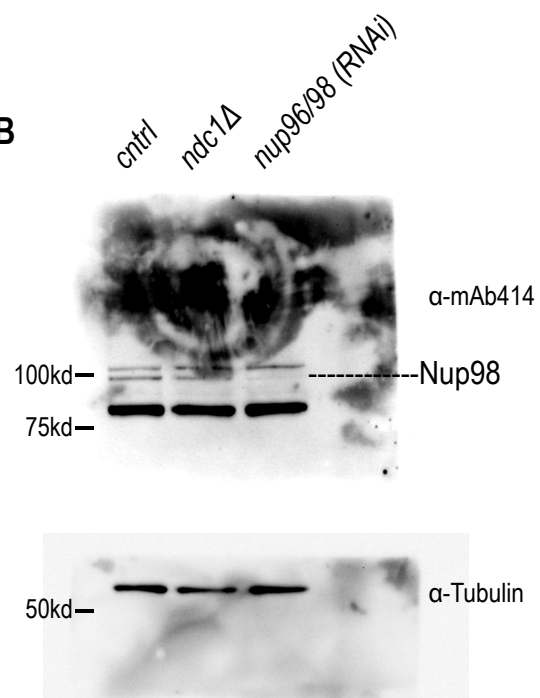**C**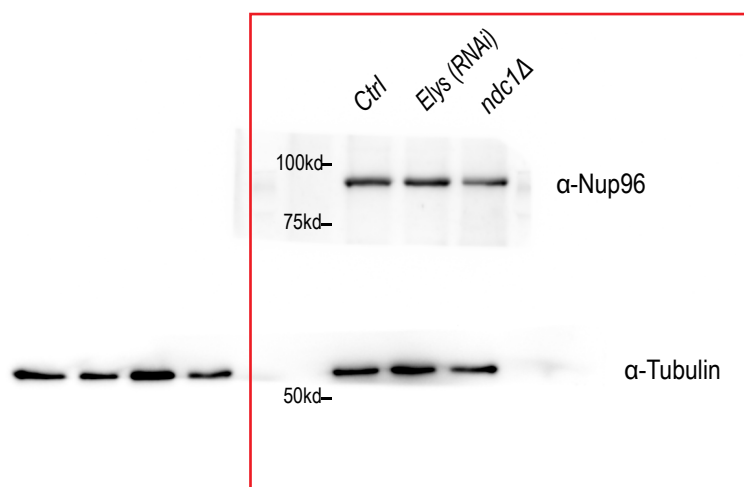**D**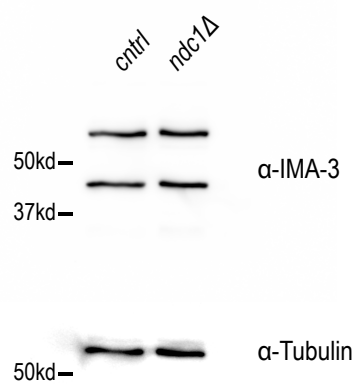

Supplement: Figure 3—figure supplement 2—source data 2. — (A) Source image for blot in B (Nup53 levels). (B) Source image for blot in D (mAb414 levels). (C) Source image for blot in E (Nup96 levels). (D) Source image for blot in F (IMA-3 levels). [file elife-75513-fig3-figsupp2-data2.pdf]
